# Development and Efficacy Determination of an Attenuated Vaccine Against Channel Catfish Virus

**DOI:** 10.3390/v18101152

**Published:** 2026-10-17

**Authors:** Suja Aarattuthodi, Lester Khoo, Nicholas Phelps, Ganesh Kumar, Kuttichantran Subramaniam, Brian Bosworth

**Affiliations:** 1 United States Department of Agriculture—Agricultural Research Service, Plant Genetics Research Unit, Columbia, MO 65211, USA; 2 Thad Cochran National Warmwater Aquaculture Center, College of Veterinary Medicine, Mississippi State University, Stoneville, MS 39762, USA; khoo@cvm.msstate.edu; 3 Department of Fisheries, Wildlife and Conservation Biology, University of Minnesota, St. Paul, MN 55108, USA; phelp083@umn.edu; 4 Thad Cochran National Warmwater Aquaculture Center, Delta Research and Extension Center, Mississippi State University, Stoneville, MS 38776, USA; gkk27@msstate.edu; 5 College of Veterinary Medicine, University of Florida, Gainesville, FL 32611, USA; kuttichantran@ufl.edu; 6 Warmwater Aquaculture Research Unit, United States-Department of Agriculture—ARS, Stoneville, MS 38776, USA; brian.bosworth@usda.gov

**Keywords:** channel catfish virus, attenuated vaccine, cell culture, protective immunity, efficacy

## Abstract

Catfish aquaculture is the largest domestic sector of the U.S. aquaculture industry. Intensive aquaculture production systems are vulnerable to infectious disease outbreaks, resulting in significant economic losses. Catfish herpesviruses cause substantial mortality in catfish fry and fingerlings. Channel catfish virus (CCV) causes acute hemorrhagic infection and mortalities in catfish during the hatchery and nursery phases of production. Considering the key role of the catfish industry in U.S. aquaculture, it is imperative to control disease-related fish losses. Vaccines are considered the most effective proactive disease management strategy against viral infections. An attenuated CCV vaccine was developed by serial subculturing (P41) of the wild-type virus in channel catfish ovary (CCO/BB) cell cultures. This attenuated vaccine was administered via immersion to channel and hybrid catfish fingerlings. Thirty days post-immunization, the immunized and non-immunized fish were exposed to wild-type CCV. The percent survival was significantly higher in the immunized channel (96%) and hybrid (98%) catfish compared to the non-immunized group, confirming the protective immunity conferred by the attenuated vaccine. Initial trials suggest that the attenuated CCV vaccine developed in this study was found to be efficacious in reducing CCV-associated mortalities in catfish fingerlings. Further trials are needed to assess the safety of the vaccine.

## 1. Introduction

Viral diseases are an ongoing challenge in several aquaculture sectors, especially in intensive aquaculture systems [1,2,3,4]. The U.S. catfish industry has consistently been proactive in adopting new production-enhancing technologies [5]. However, intensification leads to added risks of infectious disease outbreaks [6]. Viral diseases are a major concern in the hatchery and nursery phases of catfish rearing. Associated fish mortalities can affect the availability of catfish fry and fingerlings, impacting the viability of farm operations. Herpesviruses such as Ictalurid herpesvirus-1 (IcHV-1) and 2 belonging to the family *Alloherpesviridae* cause severe hemorrhagic disease and mass mortalities in fry and fingerlings of ictalurid catfish [7,8,9,10,11,12,13,14,15]. Channel catfish virus (CCV; IcHV-1) is the most significant virus affecting U.S. farm-raised catfish [7,10,11,12,16].

Channel catfish virus outbreaks are often reported during summer months when the water temperature is above 25 °C. Viral infections are particularly problematic in fish less than six months of age, with disease transmission occurring either horizontally or vertically [10,11,17,18,19,20]. The CCV disease (CCVD) outbreaks can severely impact catfish farms, with mortalities approaching 100% in some of the affected production units. CCV is highly prevalent in catfish, with the virus being endemic to most of the cultured catfish populations. According to Thompson et al. [21], the mean percent of CCV-positive channel catfish in commercial production populations in Mississippi was 64%. Survivors remain long-term carriers of the virus with the ability to transmit the virus to naïve fish [11].

Preventing CCV outbreaks at commercial catfish farms would have a great economic impact since catfish farming is the primary contributor to U.S. aquaculture, accounting for 74% of the total finfish production [22]. Currently, no effective control measures are available against CCV, and no commercial vaccines exist. Protection of the cultured fish depends on following best management practices to limit stress during hatching and the early nursery pond phase of culture. Disease prevention strategies are limited to avoidance, use of virus-free broodstock, and adoption of better management practices in hatcheries and nursery facilities [23]. Pathogen-targeted management strategies would be more effective in the control of CCV, which in turn would enhance animal health, production, and subsequently economic efficiency.

Vaccination plays a prime role in the mitigation of infectious diseases in farmed animals, including fish [6,24,25,26,27,28]. Disease prevention strategies based on the stimulation of immune factors within the host are widely accepted [29]. Fish immunization through vaccination has been practiced for more than 50 years and is considered the best disease control method that ensures environmental and economic sustainability [27]. There have been several studies attempting to leverage the host immune responses against surface proteins of the CCV particle [30,31]. However, a commercial vaccine is not yet available due to the cost of vaccine development and production, complex regulatory approval processes, difficulties in vaccine delivery, and lack of knowledge regarding the existence of multiple CCV strains. The presence of CCV in nearly all catfish-growing regions of the U.S. and the absence of an effective commercial vaccine necessitated this study. This preliminary exploratory study aimed to evaluate the efficacy of an attenuated CCV vaccine developed by serial subculturing of a wild-type virus in a catfish cell line.

## 2. Materials and Methods

### 2.1. Fish

Healthy channel catfish (*Ictalurus punctatus*) and hybrid (♀ channel catfish x ♂ blue catfish, *I. furcatus*) catfish fry at 10 days post-hatch (dph) were obtained from the USDA-ARS Warmwater Aquaculture Research Unit, Stoneville, MS. Flow-through systems were used in the study. Fish were stocked in glass aquaria of 80 L capacity with aerated water (flow rate ~1 L/min). Fish were fed daily with a diet (Rangen^®^, Buhl, ID, USA) containing 65% protein until satiation and transitioned to a 40% protein diet at 30 dph. Water quality parameters, including temperature (Traceable thermometer, Fisherbrand, Chelmsford, MA, USA), dissolved oxygen (Orion star™, Thermo scientific, Chelmsford, MA, USA), and total ammonia nitrogen (Hach, Loveland, CO, USA) were periodically monitored. All challenge protocols were approved by the Mississippi State University Institutional Animal Care and Use Committee (IACUC-19-084).

### 2.2. Cell Line and Virus

Channel catfish ovary (CCO/BB) cells (ATCC^®^ CRL-2772) [32] were used for propagating CCV. The cells were grown in Leibovitz’s L-15 medium (Corning CellGro, Manassas, VA, USA) supplemented with 10% Fetal Bovine Serum (FBS, Gibco Life Technologies, Grand Island, NY, USA). Antibiotics (100 units/mL Penicillin, 100 µg/mL Streptomycin) and antifungal agent (1 μg/mL Amphotericin B) were used to prevent bacterial or fungal contamination. A cell culture-viable virulent CCV isolate (S03-468) was obtained from the Aquatic Research and Diagnostic Laboratory (ARDL), Stoneville, MS which was used in the development of the vaccine and in the challenge trials.

### 2.3. Virus Attenuation

Filtered (0.22 µm syringe filter (Fisher Scientific, Pittsburgh, PA, USA)) CCV was inoculated onto confluent monolayers of CCO/BB cells with a volume ratio of 1:4 (virus inoculum to cell culture medium). After incubation for 1 h at 25 °C, the medium containing virus inoculum was replaced with fresh medium. The culture flasks with the cells were incubated at 25 °C and were monitored daily under an inverted microscope (Zeiss-Primovert, Carl Zeiss Microscopy LLC, White Plains, NY, USA) for CCV-specific cytopathic effects (CPEs). Once complete dissociation of the cell layer was observed, the detached cells along with the media were harvested. Virus attenuation was carried out by serial passaging following previously published protocols [33,34,35,36]. The filtered virus suspension was inoculated onto CCO/BB cells, followed by incubation at 25 °C (Passage 1; P1). Once the cells were completely dissociated from the culture surface, subsequent passages were carried out 41 times, as mentioned above. The virus obtained at P41 was selected as a candidate for vaccine evaluation. Attenuation of CCV was operationally defined as a putative reduction in virulence associated with serial passage in CCO/BB cells, as indicated by the observed changes in the timing of CPE development. The virus titer at 50% infectivity (TCID_50_) was determined at different passages to confirm virus attenuation [37].

### 2.4. Immunization of Catfish with Attenuated CCV Vaccine

**Figure 1:**
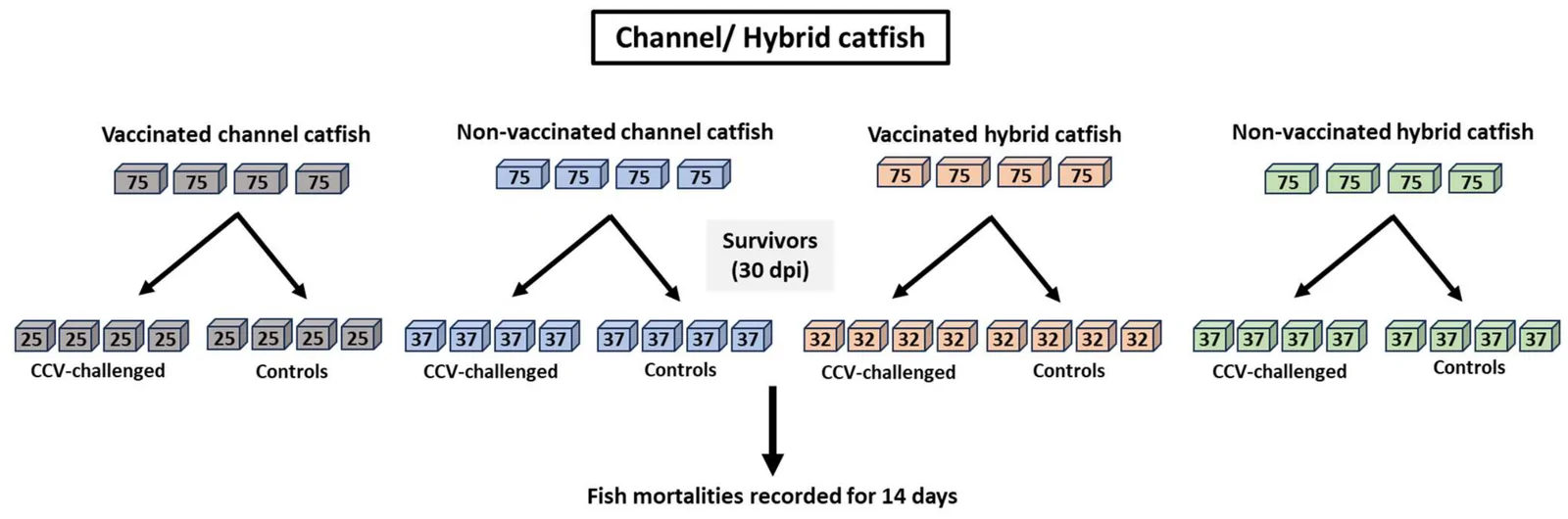
Experimental design to evaluate the efficacy of the attenuated channel catfish virus (CCV) vaccine.

Channel and hybrid catfish (1.0 ± 0.2 g; 112 dph) were stocked into 16 tanks, each containing 9 L of aerated water (n = 75 fish/tank). A schematic representation of the experimental layout is provided (Figure 1), including the time periods of immunization and subsequent challenge with wild-type CCV. Catfish fingerlings in four tanks were administered the attenuated CCV vaccine (10^4^ TCID_50_/mL) via immersion for three hours. Water flow to the tanks was cut off during the immunization and restarted thereafter. The control fish (4 tanks) were sham-vaccinated following similar water flow conditions to those of the vaccinated groups. Mortalities were monitored and recorded daily for 30 days post-vaccination, and the cause of death was confirmed by observing the CPE caused by the virus in the CCO/BB cell line.

### 2.5. Fish Challenge with Wild-Type CCV

Thirty days post-vaccination, survivors from the vaccinated and non-vaccinated treatments (Figure 1) were divided into eight tanks and four tanks were challenged with wild-type CCV (10^3.5^ TCID_50_/mL) via immersion exposure for 3 h [14]. Water flow was stopped prior to the challenge and restarted afterwards. Mortalities were monitored and recorded for 14 days post-challenge in both immunized and non-immunized groups of catfish. The cause of infection due to the virus was confirmed after inoculating the processed infected tissue extract onto healthy CCO/BB cells and observing for CPEs. DNA extracted from the virus-infected cell cultures was used for qPCR verification using CCV-specific primers [11]. Freshly dead or moribund fish were used for histopathological analysis. The percent survival of the fish was calculated to evaluate the efficacy of the attenuated CCV vaccine.

### 2.6. Histopathology

Tissue samples from the vaccinated and non-vaccinated catfish were analyzed for histopathological changes. The coelomic cavity was opened and the whole fish was fixed in 10% (*V*/*V*) neutral buffered formalin for at least 24 h, decalcified (Cal-Ex, Fisher Scientific, Fair Lawn, NJ, USA), processed, and embedded in paraffin. Tissue sections were stained with H&E (hematoxylin and eosin). Stained tissue sections were examined using a BX-50 Olympus microscope (Olympus Optical^®^, Tokyo, Japan).

### 2.7. Statistical Analysis

Fish mortality was recorded daily following challenge, and final percent survival was calculated for each experimental group at the end of the 14-day observation period. Final percent survival was analyzed using two-way ANOVA, with vaccination status (vaccinated or non-vaccinated) and catfish type (channel or hybrid) as the two factors. The analysis evaluated the effects of vaccination status and catfish type, as well as the interaction between these factors, to determine whether the effect of vaccination on survival differed between channel and hybrid catfish. Differences in means were considered statistically significant at *p* ≤ 0.05. Data are presented as mean percent survival ± standard error of the mean (SEM), calculated from the tank-level biological replicates (n). The non-challenged control groups were not included in the challenge-group statistical comparison because no mortality occurred in these groups. The cumulative survival was plotted using the daily mortality observations to illustrate survival over the 14-day post-challenge period and to provide a time-dependent assessment of mortality.

## 3. Results

### 3.1. Attenuation of the Wild-Type CCV

The wild-type virus was subcultured 41 times prior to immunization of fish. The CPEs in the virus-infected cells included syncytia, plaque formation, and destruction of cell monolayers evidenced by the detachment of cells from the culture surface. Dissociation of the cell monolayers was observed within 48–72 h after virus inoculation in earlier subcultures and extended to >5 days in later passages. However, since a direct comparative virulence assessment was not performed between the P41 virus and the parental wild-type CCV, the CPE findings do not independently establish attenuation. The virus titers were ~10^6.5^ TCID_50_/mL across passages, indicating that the observed attenuation was not associated with a proportional reduction in TCID_50_.

### 3.2. Immunization of Catfish with Attenuated CCV

Following immunization with the CCV vaccine, mortalities were observed in both channel and hybrid catfish. In channel catfish, 24.5% mortality was observed when the fish were vaccinated. Percent mortality in the vaccinated hybrid catfish was 13.5%. There were no fish mortalities in the non-vaccinated channel and hybrid catfish.

**Figure 2:**
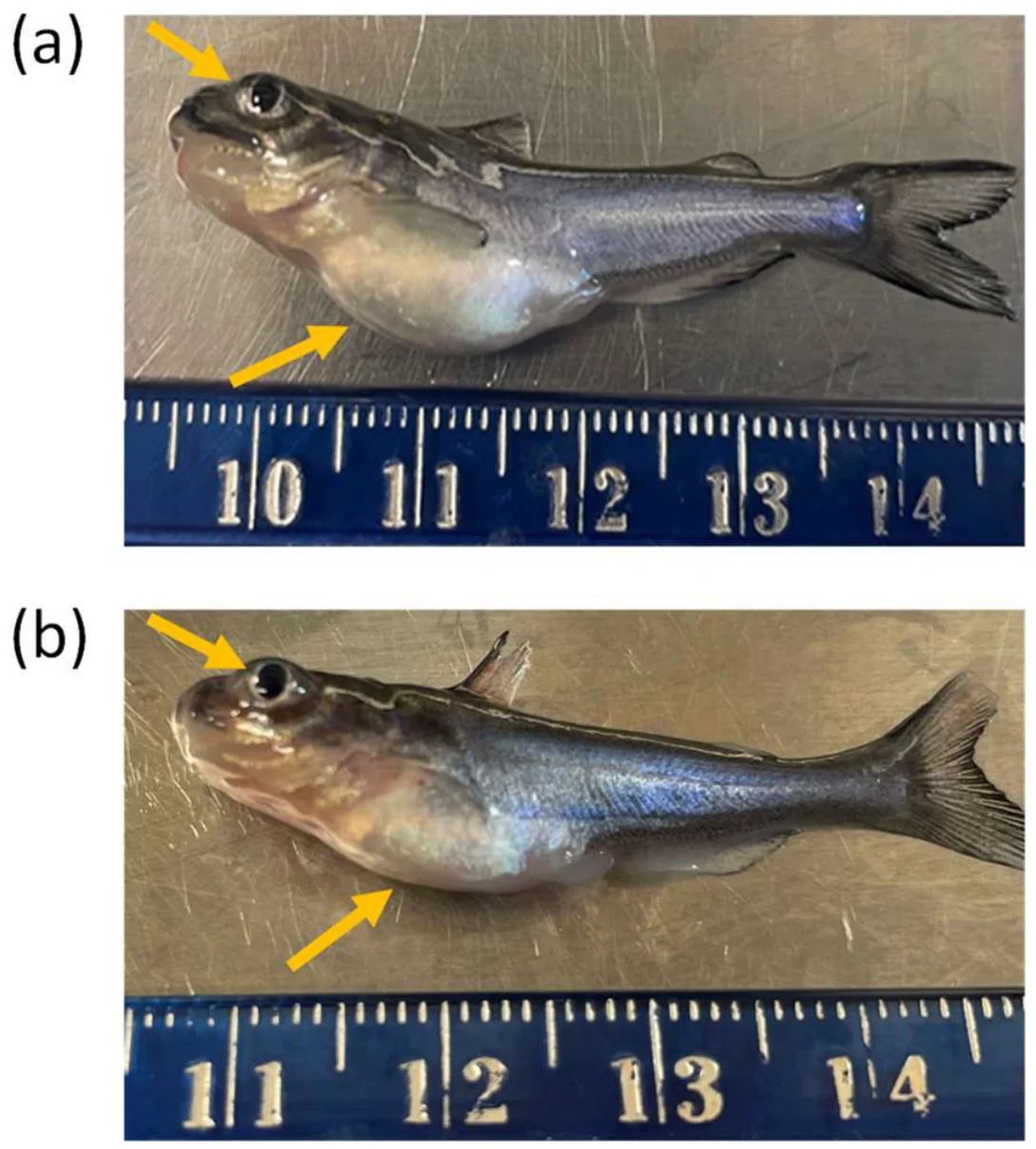
External clinical signs of channel catfish virus (CCV) infection in (**a**) channel and (**b**) hybrid catfish fingerlings. Infected fish exhibited exophthalmia and a distended abdomen (indicated by arrows).

**Figure 3:**
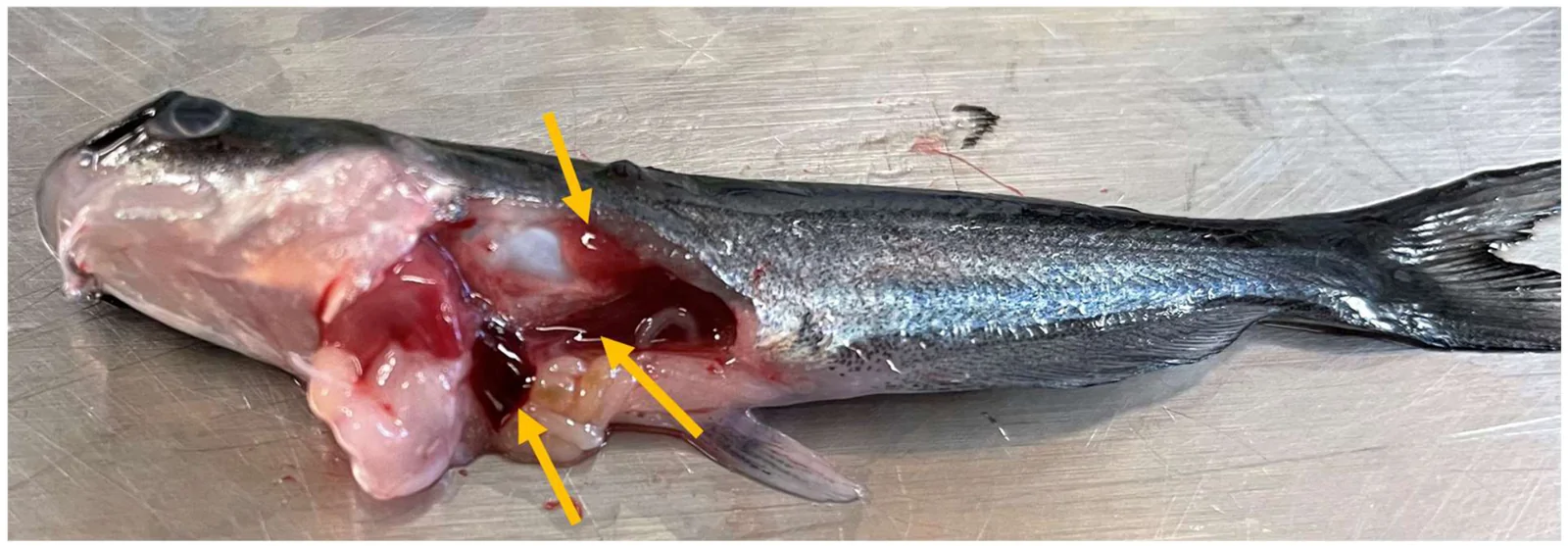
Internal clinical signs of channel catfish virus (CCV) infection in a channel catfish fingerling. Infected fish exhibited renomegaly, an enlarged, congested spleen, and ascites (indicated by arrows).

**Figure 4:**
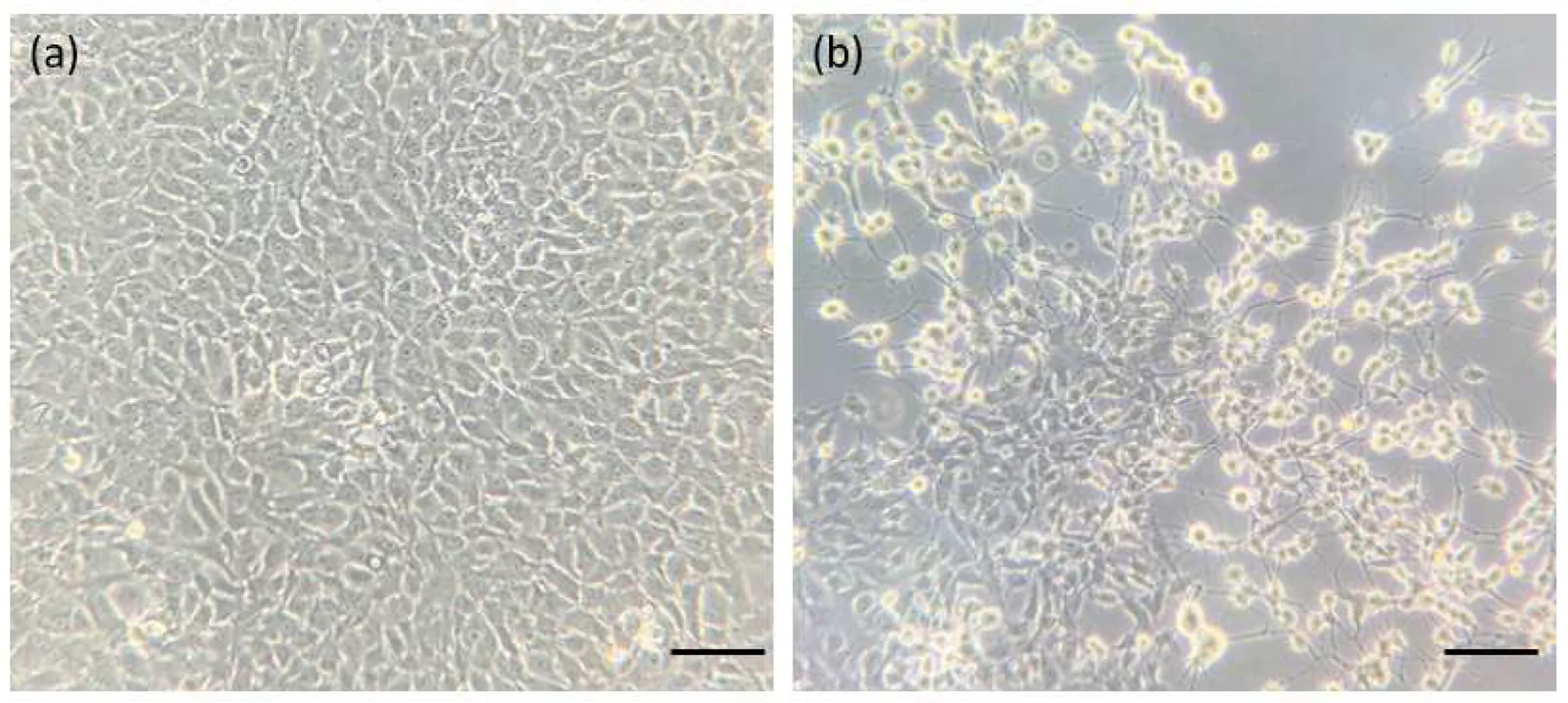
Confirmation of channel catfish virus infection by inoculating the processed tissues from the infected fish into channel catfish ovary (CCO/BB) cells. (**a**) Healthy cells. (**b**) Virus-infected cells showing dissociation of the cell monolayer within 72 h post-inoculation (Scale bar—10 µm).

The clinical signs exhibited by the moribund and diseased fish included a distended abdomen, mild to moderate exophthalmia, and hemorrhages at the base of the fins (Figure 2). The internal lesions included ascitic fluid-filled coelomic cavity, dark and congested spleen, and swollen anterior and posterior kidney (Figure 3). Spleen and kidney tissues from the freshly dead fish were processed and inoculated onto healthy CCO/BB cells and the causation of the mortalities was confirmed as CCV. The inoculated cells displayed CPEs consistent with CCV infection. The CPEs primarily involved rounding of the cells, syncytia, plaques, and complete dissociation of cell monolayers (Figure 4).

### 3.3. Challenge with Wild-Type CCV and Determination of Vaccine Efficacy

**Figure 5:**
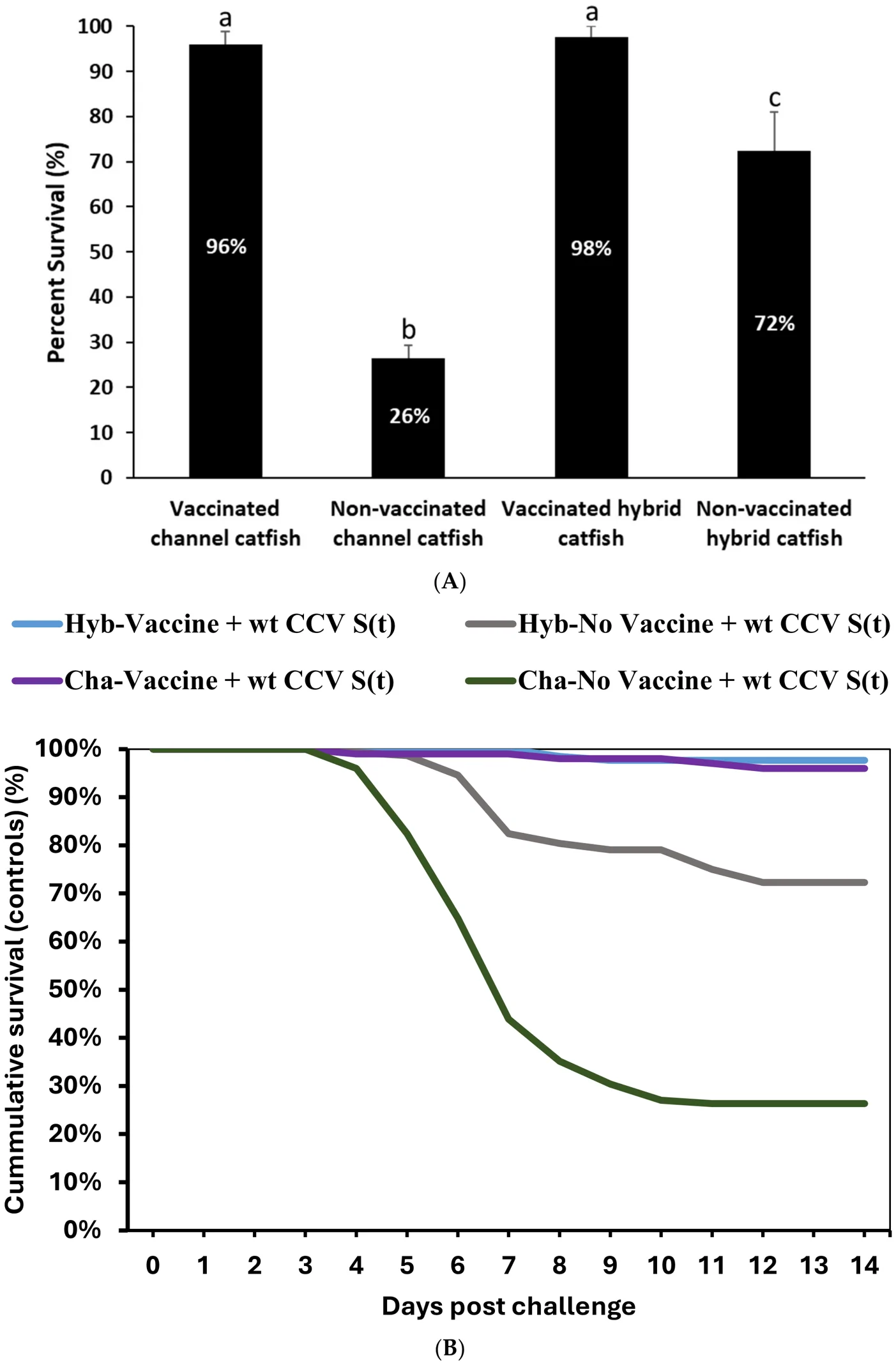
Survival of vaccinated and non-vaccinated channel and hybrid catfish following challenge with wild-type CCV 30 days post-immunization. (**A**) Final percent survival of fish following the 14-day post-challenge observation period. Data are presented as mean ± SEM. Statistical differences in the final survival were evaluated using two-way ANOVA, with vaccination status and catfish type as factors. Different letters indicate statistically significant differences (*p* ≤ 0.05). (**B**) Cumulative survival curves showing survival of fish over the 14-day period following challenge. Control groups (vaccinated/non-vaccinated channel and hybrid catfish not exposed to wild-type CCV) had 100% survival.

The percentage survival for vaccinated channel and hybrid catfish fingerlings following challenge with virulent CCV was significantly higher (96% and 98%, respectively) than their non-vaccinated counterparts (26% and 72%, respectively) (Figure 5A,B). The percent survival of non-vaccinated channel catfish was significantly lower than that of the non-vaccinated hybrid catfish. Control groups (vaccinated/non-vaccinated channel and hybrid catfish not exposed to wt CCV) had no mortalities (100% survival).

### 3.4. Histopathological Analysis

**Figure 6:**
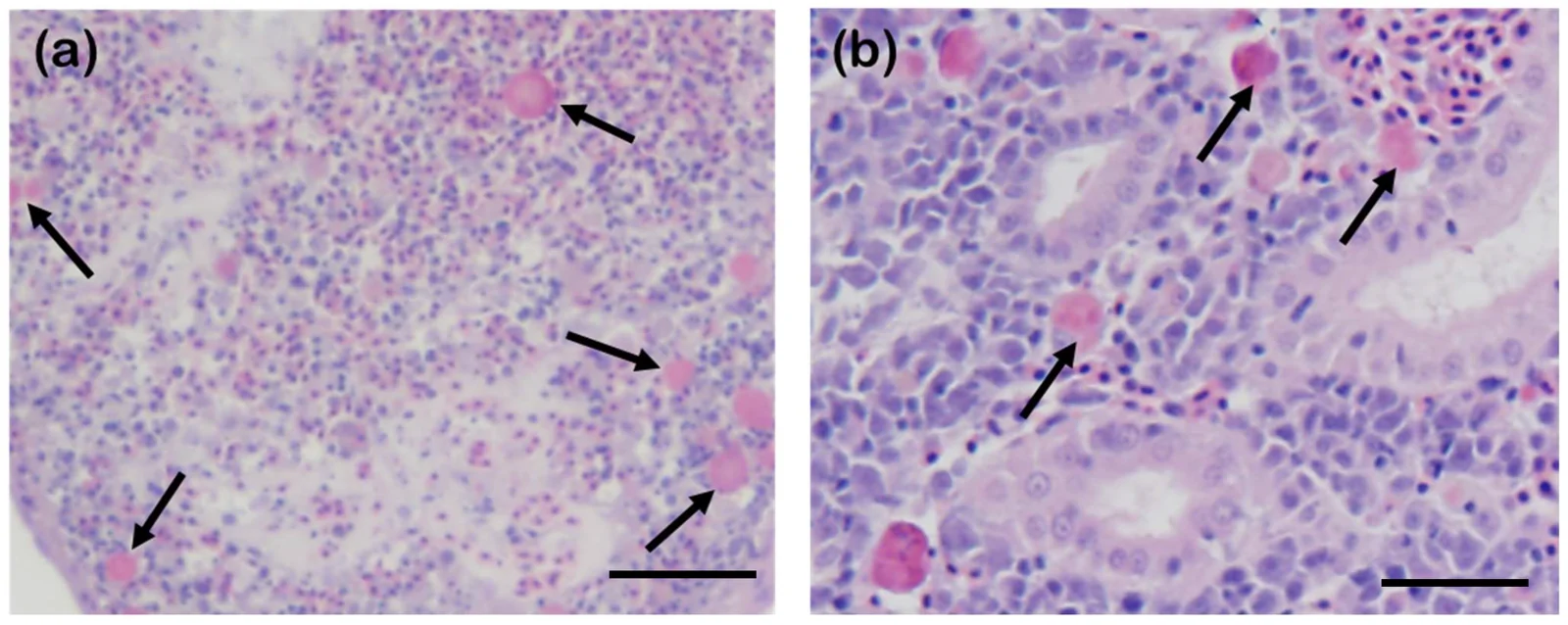
Histopathological analysis of the CCV-infected catfish tissues. (**a**) Severe erythrophagia (black arrows) in the infected spleen (Scale bar—100 µm) and (**b**) nephritis with erythrophagia in the posterior kidney were observed (Scale bar—50 µm).

The infected fish that died had lesions consistent with an experimental CCV infection while the uninfected control fish had no significant microscopic lesions. Necrotizing splenitis and nephritis were present in both the anterior and posterior kidneys with variable erythrophagia observed in these organs (Figure 6).

## 4. Discussion

Vaccination plays a key role in the mitigation of infectious diseases in farmed animals, including fish [24,26,27,28]. It is reported that catfish are efficiently immunized against CCV using vaccines and the exposed fish develop neutralizing antibodies [38,39]. The CCV antibodies can persist for over two years after exposure [38,40]. Furthermore, early-life-stage resistance against CCV was correlated to maternal transfer of antibodies that play a role in preventing disease outbreaks [20]. The CCV vaccine developed in this study was found efficacious in reducing CCV-associated mortalities in both channel and hybrid catfish fingerlings.

Attenuated viral vaccines are commonly used to control human and livestock viral diseases. Attenuated vaccines are developed by repeated passaging in cell culture, chemical or physical methods, or by gene manipulations, including deletion mutations in virulent wild-type pathogens [41,42]. While a heat-inactivated virus is poorly immunogenic, live attenuated virus vaccines (LAVs) stimulate both humoral and cellular immune responses [24,26,27,30]. LAVs simulate natural infection and are capable of multiplying inside the host with prolonged antibody production against the disease through cellular memory [43]. In the present study, the live attenuated CCV vaccine protected catfish fingerlings when exposed to wild-type CCV. The percent survival of non-vaccinated channel catfish was significantly lower than that of the non-vaccinated hybrid catfish, indicating that channel catfish are more severely affected by CCV than hybrid catfish, which was also reported by Dharan et al. [14] and Venugopalan et al. [15].

A nonpathogenic attenuated cyprinid herpesvirus-3 (CyHV-3) was isolated by serial transfer of the virus in Koi fin (KF-2) cells [35,44]. The virus harvested after 20 passages in cell culture induced disease in a small percentage of naïve fingerlings [35,44], consistent with our observations. Culture in cell lines is reported to avoid undesired recombination, complementation, and reversion to a pathogenic virus. ]. The attenuated CyHV3 virus did not cause lethal disease and efficiently protected the immunized fish against viral challenge [35,44], as observed in the current study. Bacharach and Eldar [36] developed an attenuated vaccine against tilapia lake virus (TiLV) by serial passaging of the virulent virus in a tilapia-derived cell line, which showed 55–62% protection when the hosts were challenged with the virus, similar to our study results. However, conventional cell-culture-attenuated vaccines may retain residual virulence and reversion cannot be excluded when the attenuation mechanism is unknown. The absence of genome sequencing data for the P41-attenuated CCV variant in the current study limits the ability to identify genetic changes associated with attenuation and to assess its genomic stability relative to the parental wild-type virus. Therefore, additional safety trials including in vivo back-passage/reversion assessments to determine residual virulence and genetic stability are warranted before the vaccine can be considered for further development. This is particularly relevant because some vaccine-associated mortality was observed in both channel and hybrid catfish during immunization.

The vaccine-associated mortality may reflect incomplete attenuation of the P41 virus, potentially allowing residual viral replication and disease in a subset of immunized fish. Differences in host susceptibility, together with variation in fish condition or physiological stress, may also have contributed to the observed mortality; however, these possibilities were not directly evaluated in the present study. Because CCV was recovered from tissues of freshly dead fish, further studies are needed to assess the virulence of the attenuated virus in vivo and to determine whether reducing the vaccine dose or increasing the degree of attenuation can improve safety while preserving protective efficacy.

There have been many previous attempts to develop a successful vaccine against CCV in catfish. A heat-killed CCV vaccine elicited low-level immunity in fish and was found to be ineffective in controlling infection [45]. An attenuated vaccine was developed by serial passaging of CCV in a walking catfish (*Clarias batrachus*) kidney cell line [34]. This vaccine resulted in a survival rate of 90% and was then improved by booster doses (~95% survival) [30]. Zhang and Hanson [31] attenuated CCV by deleting thymidine kinase (TK) genes. A recombinant vaccine was also found to be successful against CCV [46], where gene 50 (which encodes a secreted glycoprotein) is deleted. The attenuated CCV vaccine [34] also possesses a deletion in gene 50, which is partly responsible for the virulence of the wild-type CCV. As gene 50 deletion prevents expression of the glycoprotein, reversion to the pathogenic state and the possibility of active infection with the attenuated vaccine are avoided. A sub-unit vaccine developed from the soluble envelope proteins of CCV was capable of eliciting immune responses against CCV infection [47]. Intramuscular injection of a DNA vaccine elicited virus-neutralizing antibodies and resulted in 74–78% survival of the fish [48]. Bath vaccination of eggs and fry with a subunit vaccine and bath immunization of fingerlings with an attenuated virus vaccine are reported to be successful [49]. However, these vaccines are not licensed for commercial use in the U. S. and, therefore, at present, no commercial vaccine is available that can assure broad protection against IcHV-1 infections in the fry and fingerlings of channel and hybrid catfish.

An important observation in this study was the difference in survival between channel and hybrid catfish following immunization and wild-type CCV challenge. In non-vaccinated fish, survival was 26% in channel catfish compared with 72% in hybrid catfish, whereas survival following vaccination increased to 96% and 98%, respectively. The high survival rate in the vaccinated channel and hybrid catfish fingerlings in the present study following exposure to virulent CCV shows the potential of this attenuated vaccine to elicit immune responses in the host. Although high survival was observed in both vaccinated groups, the apparent benefit of vaccination was greater in channel catfish because of their substantially lower survival in the non-vaccinated group. This observation is consistent with previous reports indicating that channel catfish are highly susceptible to CCV, particularly during early-life stages, whereas hybrid catfish may exhibit greater resistance to CCV and other diseases relevant in catfish production [20].

The relatively higher survival of non-vaccinated hybrid catfish also indicates that the magnitude of vaccine-associated protection may depend on the susceptibility of the host population. Hybrid catfish are widely used in intensive production because of desirable production characteristics, including growth performance and resistance to several important diseases. Therefore, the smaller difference between vaccinated and non-vaccinated hybrids should not be interpreted as evidence of reduced vaccine immunogenicity without additional immunological and challenge studies. Rather, the results suggest that the potential value of vaccination may be particularly evident in more susceptible channel catfish populations, while its additional benefit in relatively more resistant hybrid catfish may depend on factors such as age, environmental conditions, viral exposure level, and the prevalence or virulence of circulating CCV strains.

The potency of the vaccine can be influenced by several internal and external factors, such as the age or size of fish, water temperature, vaccine type and method of delivery, husbandry practices, production systems, drugs and antibiotic therapy, nutritional factors, and the genetic groups. Since this efficacy trial was conducted under controlled laboratory conditions, vaccine performance and survival outcomes may vary across other experimental settings, and inter-trial variability cannot be excluded. Zhang et al. [50] reviewed that the efficacy and immunogenicity of the vaccine vary with the method of administration. Different routes of vaccine delivery have been reported to influence the growth rate, biochemical indices of the blood, and survival rate in Pike perch (*Sander lucioperca*) [51]. Vaccination through immersion is widely accepted as a safe and inexpensive route of delivery with minimal stress to fish. During immunization through immersion, the host takes up the antigens through the skin, gills, or gut [52]. The water stability and the solubility of the constituents need to be considered during vaccine development [24]. However, a higher survival rate in the immunized catfish in the current study shows that the immersion route is promising for the CCV vaccine administration.

While the study provides valuable information on the attenuated CCV vaccine, it has several limitations that should be considered when interpreting these findings. Histopathological findings in the present study were limited to moribund or dead fish. Since vaccinated fish that survived the challenge were not examined histologically, the extent of tissue-level protection and the presence of subclinical lesions in surviving fish cannot be determined. Although protection was demonstrated by survival following experimental challenge, immune parameters such as neutralizing antibody responses, cellular immune responses, or other markers of vaccine-induced immunity were not measured. Consequently, the mechanisms underlying protection and the basis for the difference in survival between channel and hybrid catfish cannot be determined from the present data. Additional studies incorporating immunological measurements, quantitative assessment of viral replication in target tissues, different age groups and genetic backgrounds, and challenge under conditions that more closely represent commercial production systems will be important for determining the broader applicability of this vaccine candidate.

The primary objective of this initial proof-of-concept study was to generate an attenuated CCV vaccine candidate through serial passage in CCO/BB cells and determine whether the resulting virus could induce protective immunity against wild-type CCV. Accordingly, the study was designed primarily to assess vaccine efficacy rather than provide a comprehensive vaccine safety assessment. The mortality observed following immunization indicates that the vaccine dose and/or degree of attenuation require further optimization. Additional safety studies including dose-dependent evaluations, direct phenotypic and molecular comparisons of the parental and attenuated viruses, and in vivo safety/back-passage assessments to determine residual virulence and genetic stability are warranted before further vaccine development. Considering the role of stressors in reducing the immunity of the fish [53,54] and in turn, the efficacy of the administered vaccines, it is also relevant to evaluate the efficacy of the developed vaccine in the presence of acute and chronic stressors.

Although vaccines and antiviral drugs hold considerable promise for the effective management of viral disease outbreaks, no effective vaccines or therapeutic options are currently available for the control of CCV infections. The attenuated CCV vaccine developed in this study significantly improved survival in catfish fingerlings, demonstrating its potential as a promising strategy for CCV disease prevention. Further studies are warranted to evaluate the immunization efficacy and protective duration of the vaccine among different age groups and to characterize the host immune responses elicited following vaccination, including the immune factors and genes involved in protection and the underlying mechanisms of vaccine-mediated immunity. In addition, evaluating the cross-protective efficacy of the attenuated CCV vaccine against blue catfish alloherpesvirus would provide valuable insight into its broader applicability and potential utility for controlling alloherpesvirus infections in catfish aquaculture.

## Data Availability

The original contributions presented in this study are included in the article. Further inquiries can be directed to the corresponding author(s).
